# RhoE Is Regulated by Cyclic AMP and Promotes Fusion of Human BeWo Choriocarcinoma Cells

**DOI:** 10.1371/journal.pone.0030453

**Published:** 2012-01-17

**Authors:** Gavin P. Collett, Xue Fang Goh, Elizabeth A. Linton, Christopher W. G. Redman, Ian L. Sargent

**Affiliations:** Nuffield Department of Obstetrics and Gynaecology, University of Oxford, Oxford, United Kingdom; Brigham and Women's Hospital, United States of America

## Abstract

Fusion of placental villous cytotrophoblasts with the overlying syncytiotrophoblast is essential for the maintenance of successful pregnancy, and disturbances in this process have been implicated in pathological conditions such as pre-eclampsia and intra-uterine growth retardation. In this study we examined the role of the Rho GTPase family member RhoE in trophoblast differentiation and fusion using the BeWo choriocarcinoma cell line, a model of villous cytotrophoblast fusion. Treatment of BeWo cells with the cell permeable cyclic AMP analogue dibutyryl cyclic AMP (dbcAMP) resulted in a strong upregulation of RhoE at 24h, coinciding with the onset of fusion. Using the protein kinase A (PKA)-specific cAMP analogue N^6^-phenyl-cAMP, and a specific inhibitor of PKA (14–22 amide, PKI), we found that upregulation of RhoE by cAMP was mediated through activation of PKA signalling. Silencing of RhoE expression by RNA interference resulted in a significant decrease in dbcAMP-induced fusion. However, expression of differentiation markers human chorionic gonadotrophin and placental alkaline phosphatase was unaffected by RhoE silencing. Finally, we found that RhoE upregulation by dbcAMP was significantly reduced under hypoxic conditions in which cell fusion is impaired. These results show that induction of RhoE by cAMP is mediated through PKA and promotes BeWo cell fusion but has no effect on functional differentiation, supporting evidence that these two processes may be controlled by separate or diverging pathways.

## Introduction

The syncytiotrophoblast layer of the human placenta is a large multinucleated epithelium forming the outer surface of the placental villi. It is in direct contact with maternal blood and is the site of key placental functions such as nutrient and gas exchange, and the synthesis of steroid and peptide hormones [1]. The formation, growth and maintenance of the syncytiotrophoblast throughout pregnancy depends on continuous fusion with underlying mononuclear villous cytotrophoblast stem cells [2]. Disturbances in fusion may be involved in pregnancy disorders such as pre-eclampsia and intra-uterine growth retardation [3], [4]; however, the mechanism by which it occurs remains poorly understood. Isolated villous cytotrophoblasts aggregate and fuse in vitro to form multinucleated syncytiotrophoblast [5] and this is enhanced by treatment with cAMP, or with agents which increase intracellular cAMP levels. A number of proteins have been implicated in the fusion process, including envelope proteins derived from human endogenous retroviruses (HERVs) [6] and their receptors [7], [8], glial cells missing 1 (GCM1) [9], connexin 43 [10] and ADAM proteins [6].

The Rho family of Ras-like GTPases comprises at least 23 signalling molecules involved in numerous cellular processes, including proliferation, adhesion, migration and differentiation [11], many of which involve regulation of the actin cytoskeleton [12]. Most of these proteins are regulated by switching between an active GTP-bound form and an inactive GDP-bound form, controlled by guanine nucleotide exchange factors (GEFs) and GTPase-activating proteins (GAPs) respectively [11]. Several studies have described a role for Rho GTPases in trophoblast processes such as cell migration [13] and cytoskeletal reorganization [14] but no data have been reported on any possible role in human cytotrophoblast fusion.

RhoE/Rnd3 is a member of the Rnd subfamily of Rho GTPases, which also comprises Rnd1 and Rnd2 [15]. Unlike other Rho GTPases, the members of this subfamily lack intrinsic GTPase activity, do not bind GEFs and GAPs and therefore exist predominantly in a constitutively active GTP-bound state. Hence, their activity and function within the cell is regulated by their expression level and localization. RhoE has been shown to regulate cytoskeletal reorganisation and cell motility through inhibition of RhoA activity [16], and plays a role in processes such as cell proliferation and cell cycle progression [17], [18], apoptosis [19] and differentiation [20]. These various functions of RhoE appear to be cell type- and context-dependent. A possible role for RhoE in cell fusion has been demonstrated by the finding that, in myoblasts, RhoE expression increases until the onset of cell fusion, and this upregulation is required for the inhibition of RhoA and ROCK1 activities and subsequent myoblast fusion [21]. In this study we report for the first time that RhoE plays a role in human cytotrophoblast fusion using the BeWo choriocarcinoma cell line, a well characterised model which shares important properties with freshly isolated human villous cytotrophoblasts, most significantly the ability to fuse and form large multinucleated syncytia [22]. We show that RhoE is upregulated by cyclic AMP via activation of protein kinase A, and that knockdown of RhoE by RNA interference inhibits cell fusion. Finally, we show that RhoE upregulation is attenuated under hypoxic conditions in which cell fusion is impaired.

## Materials and Methods

### Ethics Statement

This study was approved by the Oxfordshire Research Ethics Committee C.

### Reagents

Dibutyryl cyclic AMP (dbcAMP) was obtained from Sigma, UK. The PKA-selective cAMP analog N^6^-phenyl-cAMP (Phe) was obtained from Biolog Life Science Institute, Germany. PKA inhibitor 14–22 amide (PKI) was purchased from Merck, UK.

### Cell culture

BeWo cells and JEG-3 cells, both obtained from the European Collection of Cell Cultures (Porton Down, UK), were cultured in full growth medium (Dulbecco's modified Eagle's medium/Ham's F12 supplemented with 2 mM l-glutamine, 100 IU/ml penicillin, 100 µg/ml streptomycin (Sigma) and 10% (v/v) fetal calf serum (Serum Laboratories International)). Cells were grown as a monolayer at a density of 10^7^ cells per 75 mm^2^ flask at 37°C in 95% air and 5% CO_2_, with medium changed every 48 h. For passages, cells were detached with trypsin/EDTA (Life Technologies) at 37°C, then washed in complete culture medium and replated.

### Isolation of human primary villous cytotrophoblasts

Term placentae (n = 3) were obtained with informed consent after delivery by elective caesarean section in the John Radcliffe Hospital, Oxford. Women with medical complications or previous perinatal deaths were excluded. Only singleton pregnancies without fetal abnormality or fetal growth restriction, i.e. >10th centile, and ≥37 weeks gestation were included. Villous cytotrophoblasts were prepared as described previously [23]. After digestion partially pure cytotrophoblasts were isolated using a 5–70% Percoll gradient (Amersham Biosciences UK). The fraction between densities of 1.042 g and 1.068 g was aspirated, washed with MEM-F (Minimum Essential Medium, Invitrogen, UK) containing 1% antibiotic and antimycotic solution (Sigma) and 10% fetal calf serum and pooled, before cells were counted. Cells were purified further by negative selection using immunomagnetic beads (Dynabeads pan mouse IgG, Invitrogen) coupled to antibodies raised against MHC class I (clone W6/32; Serotec, UK), to remove non-trophoblast cells, and placental alkaline phosphatase (PLAP) (NDOG2 [23]) to remove syncytial fragments. Cell purity was analysed flow cytometrically as described previously [23].

### siRNA transfection

Cells were plated at 7.5x10^4^ cells/ml into 24-well plates and transfected immediately with RhoE siRNA (Hs_RND3_3, cat. No. SI04286702, Qiagen) or a non-silencing negative control siRNA (Allstars negative control, Qiagen) using Hiperfect transfection reagent (Qiagen) following protocols provided by the manufacturer. After 48h of incubation the cells were either harvested for protein extraction and immunoblotting or treated as described below for immunocytochemistry and analysis of cell fusion.

### Immunoblotting

Cells were washed with PBS and lysed directly into SDS–PAGE loading buffer. Protein concentration was determined using a BCA protein assay kit (Pierce). Proteins (5 µg) were resolved by SDS–PAGE and transferred to PVDF membrane. Membranes were then incubated in blocking buffer (Tris-buffered saline with 5% BLOTTO (Santa Cruz) and 0.1% Tween) for 45 minutes at room temperature then incubated with the appropriate primary antibodies in blocking buffer overnight at 4°C. Reactions were visualized by using a suitable secondary antibody conjugated to horseradish peroxidase (Dako) in blocking buffer at room temperature for 2 hours and an enhanced chemiluminescence system (Pierce). Primary antibodies used were: anti-RhoE (#3664, Cell Signaling Technology, 1∶000), anti-β-actin (#ab6276, Abcam, 0.15 µg/ml), anti-hCG (#ab14301, Abcam, 1 µg/ml) and anti-placental alkaline phosphatase (NDOG2 [23], 1 µg/ml).

### Immunocytochemistry

Cells for desmosomal protein staining were fixed with ice-cold methanol and stored at 4°C in PBS until processing. Cells were permeabilised by incubation in PBS containing 0.5% Triton-X 100 (Sigma) for 10 min at room temperature. All cells were blocked for 1h at room temperature with PBS containing 10% normal human serum and 0.1% Tween-20, then incubated overnight at 4°C with a primary antibody to anti-desmosomal protein (#D1286, Sigma, 10 µg/ml), diluted in blocking buffer before washing (2×5 min) in PBS. Negative controls comprised mouse IgG alone. Cells were next stained with Alexa Fluor 488 conjugated secondary antibody (Invitrogen) in blocking buffer, the nuclei stained with Hoechst 33342 (1 µg/ml in PBS; Invitrogen) and washed (2×5 min) and stored in PBS. They were examined using a Leica DMIRE2 inverted fluorescence microscope and photographed using a Hamamatsu Orca monochrome camera and Simple PCI software (C Imaging).

### Cell fusion analysis

Cells were induced to fuse by replacing full growth medium with Dulbecco's modified Eagle's medium containing 2.5% (v/v) fetal calf serum and 1mM dibutyryl cyclic AMP, as described previously [24]. At the indicated time points the cells were washed with PBS and fixed in methanol at −20°C for 10 minutes. Intercellular boundaries were then visualized by immunocytochemical staining with an antibody to desmosomal protein as described above. Ten random fields containing approximately 300 nuclei each were photographed for subsequent analysis. Composite images of Hoechst 33342 stained nuclei and Alexa Fluor 488 stained desmosomal protein were made, then all nuclei were counted and the percentage of nuclei contained within multinucleated (>2 nuclei) syncytia was calculated, as described previously [24].

### Statistical analysis

The data presented represent mean ± SEM of at least three separate experiments. Differences between treatment groups were analysed by ANOVA and a *P*-value of<0.05 was considered to be statistically significant.

## Results

### RhoE expression is upregulated by cyclic AMP in BeWo cells and is associated with cell fusion

**Figure 1:**
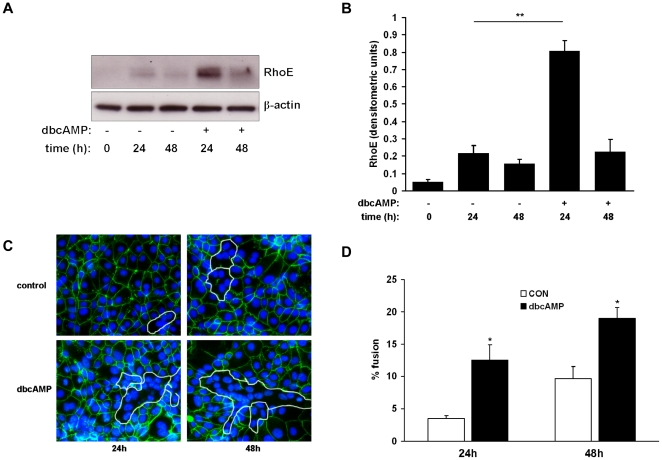
Effect of cyclic AMP on RhoE expression and fusion in BeWo cells. BeWo cells were treated with or without 1mM dbcAMP and studied at the indicated times. Cell lysates were made and expression of RhoE and β-actin was assessed by immunoblotting (A) and densitometric analysis of blots (B). Cells were fixed, immunostained for desmosomal protein (green) and counterstained with Hoechst 33258 (blue) (C) and cell fusion was quantified (D) as described in Materials & Methods. Results are presented as mean ± SEM for three separate experiments. *p<0.05, **p<0.01 compared with control (determined by ANOVA).

**Figure 2:**
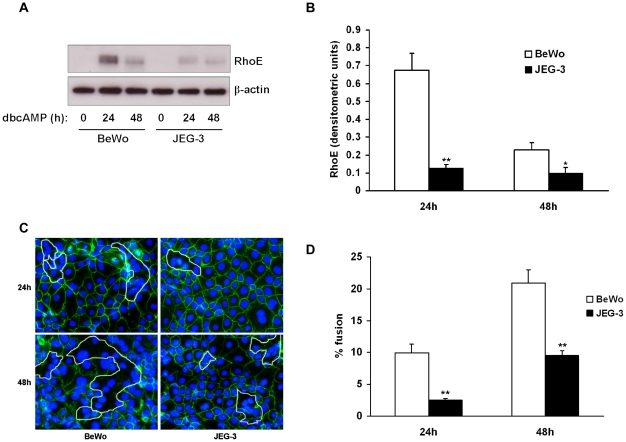
Attenuation of RhoE expression in response to cyclic AMP in JEG-3 cells. BeWo or JEG-3 cells were treated with 1mM dbcAMP. At the indicated times cell lysates were made and expression of RhoE and β-actin was assessed by immunoblotting (A) with densitometric analysis of RhoE expression normalised to β-actin expression (B). Cells were fixed, immunostained for desmosomal protein (C) and cell fusion was quantified (D) as described in Materials & Methods. Results are presented as mean ± SEM for three separate experiments. *p<0.05, **p<0.01 compared with BeWo cells (determined by ANOVA).

BeWo cells fuse in response to treatment with cyclic AMP [24], [25]. Here we sought to determine the effect of the cell-permeable cyclic AMP analogue dibutyryl cyclic AMP (dbcAMP) on RhoE expression in BeWo cells. Confluent cells were treated with low-serum medium in the presence or absence of dbcAMP at 1mM, a concentration which has previously been shown to induce cell fusion [24]. RhoE expression was then detected by immunoblotting. Treatment with dbcAMP significantly increased RhoE expression after 24h compared with control (Figs. 1A and B). To confirm stimulation of cell fusion by dbcAMP, intercellular boundaries were visualised by labelling desmosomal protein. Fusion was then quantified by calculating the percentage of nuclei contained within multinucleated syncytia. As expected, treatment with dbcAMP significantly increased fusion compared to control (12.5% vs 3.5% and 19.0% vs 9.6% at 24h and 48h respectively, Figs. 1C and D). We then assessed the effect of dbcAMP on RhoE expression in JEG-3, a human choriocarcinoma cell line which undergoes less fusion in response to cAMP compared to BeWo [26]. In JEG-3 cells, both RhoE upregulation (Figs. 2A and 2B) and intercellular fusion (Fig. 2C) were significantly attenuated in response to dbcAMP treatment compared to BeWo cells. Taken together, these results suggested a correlation between RhoE expression and cell fusion.

### Upregulation of RhoE by cyclic AMP is mediated via protein kinase A

**Figure 3:**
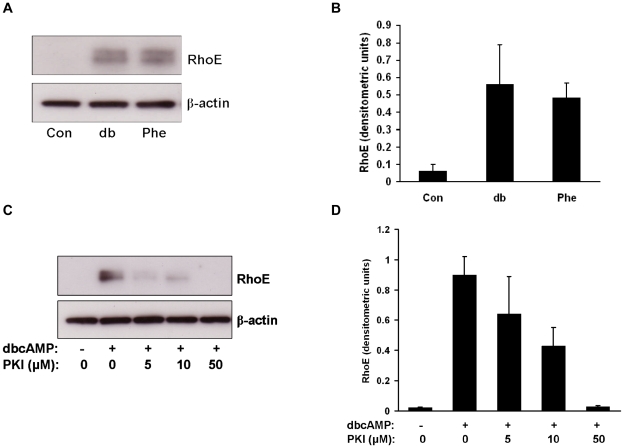
Upregulation of RhoE by cyclic AMP is mediated through protein kinase A. BeWo cells were treated with 1mM dbcAMP or PKA-selective cAMP analogue (Phe) for 24h. Cell lysates were made and expression of RhoE and β-actin was assessed by immunoblotting (A) with densitometric analysis of RhoE expression normalised to β-actin expression (B). BeWo cells were pretreated with the specific PKA inhibitor PKI or vehicle for 1h then treated with 1mM dbcAMP in the presence or absence of PKI. After 24h cell lysates were made and expression of RhoE and β-actin was assessed by immunoblotting (C) with densitometric analysis of RhoE expression normalised to β-actin expression (D). Representative blots from three separate experiments are shown.

Cyclic AMP can exert its effects by protein kinase A (PKA)-dependent and -independent mechanisms [27]. To explore the role of PKA in the upregulation of RhoE by cyclic AMP we treated BeWo cells with a PKA-selective cAMP analog, N^6^-phenyl-cAMP (Phe), for 24h and assessed RhoE expression by immunoblotting. Treatment with Phe resulted in an induction of RhoE expression equal to that given by dbcAMP treatment (Fig. 3A). We then investigated the effect of the specific PKA inhibitor 14–22 amide (PKI) on dbcAMP-induced RhoE expression. BeWo cells were pretreated with PKI or vehicle for 1h then incubated with dbcAMP with or without PKI for 24h and assessed for RhoE expression. PKI inhibited dbcAMP upregulation of RhoE (Fig. 3B). These data show that RhoE upregulation by cAMP is mediated through activation of PKA.

### RhoE knockdown inhibits BeWo cell fusion

**Figure 4:**
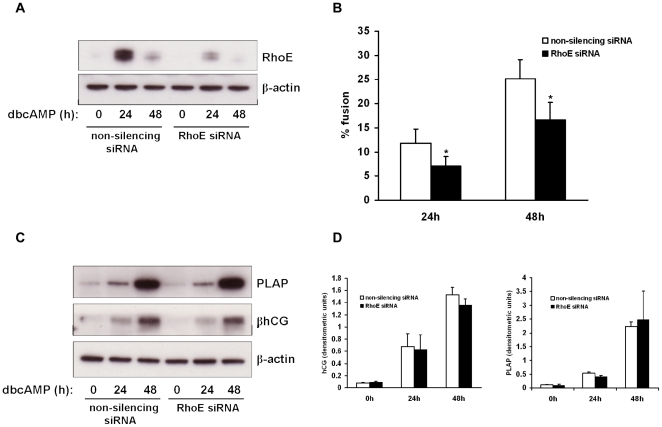
Effect of RhoE knockdown on BeWo cell fusion and differentiation. BeWo cells were transfected with 50nM RhoE siRNA or a non-silencing control then treated with 1mM dbcAMP and studied at the time points indicated. Cell lysates were made and expression of RhoE and β-actin was assessed by immunoblotting (A). Cells were also fixed and immunostained for desmosomal protein and cell fusion quantified (B) as described in Materials & Methods. Cell lysates were also studied for expression of PLAP, β-hCG and β-actin by immunoblotting (C) with densitometric analysis normalised to β-actin expression (D). Results are presented as mean ± SEM for four separate experiments. *p<0.05 compared with non-silencing control (determined by ANOVA).

Since we found that BeWo cell fusion was associated with an upregulation of RhoE, we hypothesised that RhoE may play an active role in driving this process. To test this hypothesis we used RNA interference to knock down RhoE expression. BeWo cells were transfected with siRNA duplexes targeted to human RhoE mRNA (RhoE siRNA) or a non-silencing control which has no homology to any known mammalian gene, and then treated with dbcAMP to stimulate RhoE expression. RhoE was efficiently downregulated by transfection with RhoE siRNA, as demonstrated by an immunoblot of BeWo cell lysates following dbcAMP treatment (Fig. 4A). Levels of β-actin were unaffected by transfection with either RhoE or non-silencing siRNA. Densitometric analysis revealed that transfection with RhoE siRNA resulted in a 73% knockdown of RhoE expression after 24h dbcAMP treatment compared with the non-silencing control.

We next examined the effect of RhoE knockdown on BeWo cell fusion. Cells were transfected with RhoE or control siRNA then treated with low-serum medium containing 1mM dbcAMP. RhoE siRNA-transfected cells showed a significant decrease in the percentage of nuclei contained in syncytia compared with cells transfected with non-silencing control (7.0% vs 11.9%, and 16.6% vs 25.2%, at 24h and 48h respectively; Fig. 4B). We then determined whether RhoE downregulation affected the expression of hCG and placental alkaline phosphatase (PLAP), two markers of biochemical differentiation of BeWo cells. Expression of both these proteins was induced by dbcAMP treatment but their levels were unchanged in RhoE siRNA-transfected cells compared to non-silencing control following dbcAMP treatment, as assessed by immunoblotting (Fig. 4C). These results suggest that RhoE plays a role in BeWo cell fusion, but is dispensible for the induction of some proteins associated with biochemical differentiation.

### RhoE upregulation by cyclic AMP is inhibited by hypoxia

**Figure 5:**
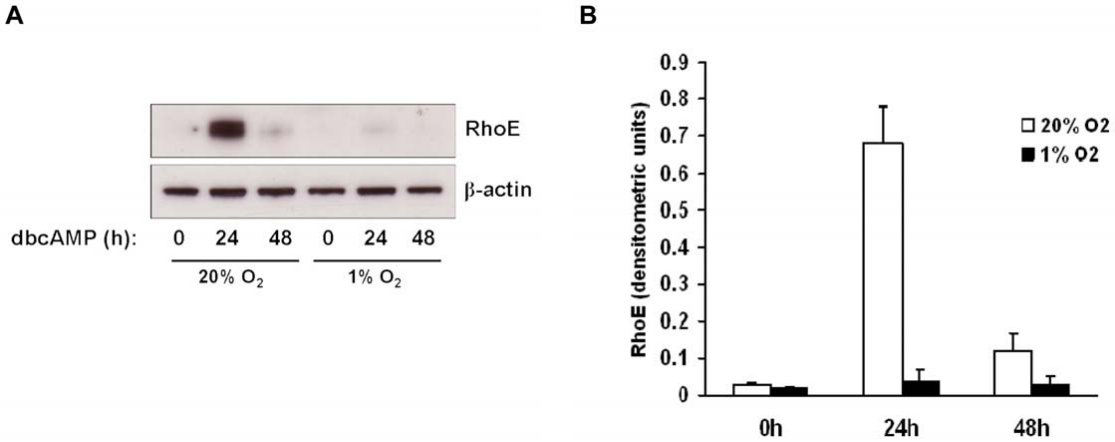
Effect of hypoxia on cAMP-induced RhoE expression. BeWo cells were cultured at 20% O_2_ (normoxia) or 1% O_2_ (hypoxia) for 24h, then treated with 1mM dbcAMP at 20% or 1% O_2_ for a further 24h. Cell lysates were made and RhoE and β-actin expression was assessed by immunoblotting (A) with densitometric analysis of RhoE expression normalised to β-actin expression (B). A representative blot from three separate experiments is shown.

It has long been known that fusion of cytotrophoblasts is inhibited under hypoxic conditions [28]. We proposed that, since RhoE plays a role in BeWo cell fusion, its induction by dbcAMP may be impaired under hypoxia. To test this, cells were grown to confluence and cultured at 20% O_2_ (normoxia) or 1% O_2_ (hypoxia) for 24h, then treated with 1mM dbcAMP at 20% or 1% O_2_ for a further 24h. RhoE expression was then assessed by immunoblotting. Although 6.5–8.6% O_2_ is regarded as placental normoxia [29], we used 20% O_2_ as normoxic conditions in these experiments as BeWo cells have been adapted in culture to grow at 20% O_2_ and thus this represents normoxia for these cells. Figure 5 shows that the induction of RhoE by dbcAMP at 24h was reduced in hypoxic conditions compared to normoxia. These data suggest that the inhibition of BeWo cell fusion under hypoxia may be due, at least in part, to impaired RhoE induction.

### RhoE is expressed in primary human villous cytotrophoblasts

**Figure 6:**
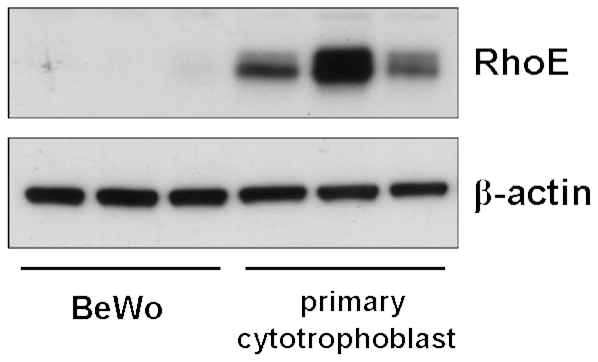
RhoE is expressed in primary human villous cytotrophoblasts. Lysates were prepared from BeWo cells or freshly isolated primary human villous cytotrophoblasts (three separate preparations) and assessed for RhoE and β-actin expression by immunoblotting.

Since RhoE plays a role in BeWo cell fusion, we addressed the relevance of these observations to normal human trophoblast by assessing the expression of RhoE in isolated primary human villous cytotrophoblasts. We found that RhoE was strongly expressed in these cells, which fuse spontaneously in culture [5], compared with the very weak expression in untreated, non-fusing BeWo cells (Fig. 6), suggesting that RhoE may play a role in normal human trophoblast functions, including fusion.

## Discussion

In this study we demonstrate for the first time that RhoE is regulated by cyclic AMP and is involved in the cyclic AMP-mediated fusion of the BeWo human choriocarcinoma cell line. These results suggest that RhoE may play an important role in the regulation of trophoblast fusion in normal and/or pathological pregnancies.

Several members of the Rho GTPase family, such as RhoA, Rac1 and cdc42, have been described in human cytotrophoblasts and shown to play a role in their migration [13]. However, a role for members of the Rho family of proteins in cytotrophoblast fusion has not been reported to date. In myoblasts, RhoE is required for intercellular fusion resulting in the formation of myotubes. In these cells RhoE expression increases until the onset of fusion before returning to its basal level once fusion is under way [21]. To date, no data exist on the expression of RhoE in primary human cytotrophoblasts or in the BeWo cell line, a well-characterised model of human cytotrophoblast differentiation and fusion [22]. Given its role in myoblast fusion, we hypothesised that RhoE may also be expressed in BeWo cells and play a role in their fusion.

Cyclic AMP treatment has long been known to promote fusion of both primary human cytotrophoblasts [30] and BeWo cells [24], [25], and to upregulate several proteins involved in fusion, including syncytin-1 [31], syncytin-2 [32], MFSD2A [33] and CD9 [34]. In the present study, treatment of BeWo cells with the cell-permeable cAMP analogue dbcAMP led to an upregulation of RhoE, concomitant with an increase in cell fusion. Since RhoE lacks intrinsic GTPase activity and is not regulated by GEFs and GAPs, control of its expression level, together with post-translational modification, is an important mechanism for the regulation of its function within the cell. A number of different stimuli have been shown to induce RhoE expression via transcriptional regulation, including chemotherapeutic agents [35], ultraviolet irradiation [36] and estradiol [37]. Phosphorylation of RhoE by ROCK1 and PKCα also upregulates its expression by increasing its stability through prevention of proteasomal degradation [38], [39].

To our knowledge, this is the first report showing upregulation of RhoE by cAMP. Interestingly, induction of RhoE by dbcAMP was significantly impaired in JEG-3 cells compared to BeWo cells. Although some studies have reported that JEG-3 cells are non-fusogenic [40], [41], we, in this study, and others [26] have shown that they do undergo some fusion in response to cAMP but at a significantly reduced level compared to BeWo cells. These results led us to hypothesise that this correlation between cAMP-induced RhoE expression and subsequent fusion may indicate a direct role for RhoE in BeWo cell fusion. In support of this hypothesis we found that knockdown of RhoE expression by RNA interference resulted in a significant inhibition of BeWo cell fusion. Although significant, the reduction in fusion we observed following RhoE depletion was relatively modest. This may be due to upregulation of other Rnd subfamily members in response to dbcAMP, or may indicate the concurrent activation of alternative, Rnd subfamily-independent pathways which also promote cell fusion. However, it may also be the result of insufficient knockdown; although immunoblot analysis revealed 73% knockdown, there was still clearly some upregulation of RhoE in response to dbcAMP which may have been sufficient to induce some fusion. As in myoblasts [21], RhoE expression is maximal at the onset of fusion and then declines at later time points despite a further increase in fusion, suggesting that RhoE is required relatively early in the fusion pathway and is no longer required once fusion is complete.

Interestingly, expression of two differentiation-associated proteins, hCG and PLAP, was unaffected by RhoE knockdown, suggesting that RhoE is required for fusion but may be dispensible for biochemical differentiation. This supports the findings of recent work which showed that treatment of BeWo cells with a PKA inhibitor, H-89, led to an inhibition of fusion but had no effect on hCG expression [42], and suggests that trophoblast fusion and functional differentiation may be controlled by separate or diverging pathways. Similarly, in myoblasts knockdown of RhoE impairs myotube formation but has no effect on the expression of myogenin and troponin T, two muscle-specific proteins [21].

Cyclic AMP can exert its effects through activation of protein kinase A, exchange protein directly activated by cAMP (Epac) and cAMP-gated ion channels. Using a PKA-specific cAMP analogue and a pharmacological PKA inhibitor we found that upregulation of RhoE by cAMP in BeWo cells is mediated through the PKA pathway. Activation of PKA has been shown to promote trophoblast fusion [30]. Many effects of PKA are elicited via its phosphorylation of cyclic AMP response element binding protein (CREB) at Ser^133^ and subsequent transcription of target genes [27]. In BeWo cells PKA-induced CREB phosphorylation leads to increased transcription of GCM1 [43], [44], a transcription factor which is essential for trophoblast fusion. Furthermore, PKA stimulates CREB-binding protein-mediated acetylation of GCM1, resulting in an elevation of its activity [45]. Therefore, it is tempting to speculate that cAMP-PKA signalling may upregulate RhoE expression through increased expression and/or activation of GCM1. This notion is supported by our finding that upregulation of RhoE by cAMP is attenuated under hypoxic conditions, since GCM1 activity is decreased in trophoblasts in hypoxia as a result of enhanced degradation [46], and leads us to speculate that this attenuation of RhoE expression may form part of the mechanism by which BeWo cell fusion is inhibited in hypoxia [47]. However, we cannot rule out the possibility that RhoE may be upregulated by direct phosphorylation by PKA, leading to increased stability in a manner similar to that elicited by PKCα and ROCK1.

The mechanism by which RhoE regulates BeWo cell fusion remains to be determined. In myoblasts, upregulation of RhoE leads to a p190RhoGAP-mediated inhibition of RhoA and ROCK1 activities, which are required for fusion to take place. This may be mediated through M-cadherin which is upregulated and accumulates at cell-cell contact sites in a RhoE-dependent manner [21]. There are no reports documenting RhoA or ROCK1 activities during trophoblast fusion but it has been shown that fusion is inhibited in primary human cytotrophoblasts transfected with antisense oligonucleotides specific for cadherin-11 [48]. Therefore it may be that RhoE may promote fusion in BeWo cells by increasing cadherin-11 expression and/or localisation, possibly through a mechanism involving modulation of RhoA and ROCK1 activities. These are themes which will be explored in future work.

In conclusion, the present study has identified RhoE as a new target for cAMP-PKA signalling and a mediator of fusion in BeWo cells. Our finding that RhoE is strongly expressed in isolated primary human cytotrophoblasts indicates that further studies will be required to fully understand the role of RhoE in trophoblast function.

## References

[1] Benirschke K, Kaufmann P (2000). Pathology of the human placenta..

[2] Midgley AR, Pierce GB, Deneau GA, Gosling JRG (1963). Morphogenesis of syncytiotrophoblast in vivo: an autradiographic demonstration.. Science.

[3] Potgens AJ, Schmitz U, Bose P, Versmold A, Kaufmann P (2002). Mechanisms of syncytial fusion: a review.. Placenta.

[4] Ruebner M, Strissel PL, Langbein M, Fahlbusch F, Wachter DL (2010). Impaired cell fusion and differentiation in placentae from patients with intrauterine growth restriction correlate with reduced levels of HERV envelope genes.. J Mol Med.

[5] Kliman HJ, Nestler JE, Sermasi E, Sanger JM, Strauss JF (1986). Purification, characterization, and in vitro differentiation of cytotrophoblasts from human term placentae.. Endocrinology.

[6] Rote NS, Chakrabarti S, Stetzer BP (2004). The role of human endogenous retroviruses in trophoblast differentiation and placental development.. Placenta.

[7] Huppertz B, Bartz C, Kokozidou M (2006). Trophoblast fusion: fusogenic proteins, syncytins and ADAMs, and other prerequisites for syncytial fusion.. Micron.

[8] Liang CY, Wang LJ, Chen CP, Chen LF, Chen YH (2010). GCM1 regulation of the expression of syncytin 2 and its cognate receptor MSFD2A in human placenta.. Biol Reprod.

[9] Yu C, Shen K, Lin M, Chen P, Lin C (2002). GCMa regulates the syncytin-mediated trophoblastic fusion.. J Biol Chem.

[10] Frendo JL, Cronier L, Bertin G, Guibourdenche J, Vidaud M (2003). Involvement of connexin43 in human trophoblast cell fusion and differentiation.. J Cell Sci.

[11] Bustelo XR, Sauzeau V, Berenjeno IM (2007). GTP-binding proteins of the Rho/Rac family: regulators, effectors and functions in vivo.. Bioessays.

[12] Lee SH, Dominguez R (2010). Regulation of actin cytoskeleton dynamics in cells.. Mol Cells.

[13] Han J, Li L, Hu JY, Lu LL, Zheng YR (2010). Epidermal growth factor stimulates human trophoblast cell migration through RhoA and RhoC activation.. Endocrinology.

[14] Parast MM, Aeder S, Sutherland AE (2001). Trophoblast giant cell differentiation involves changes in cytoskeleton and cell motility.. Dev Biol.

[15] Riou P, Villalonga P, Ridley AJ (2010). Rnd proteins: multifunctional regulators of the cytoskeleton and cell cycle progression.. Bioessays.

[16] Talens-Visconti R, Peris B, Guerri C, Guasch RM (2010). RhoE stimulates neurite-like outgrowth in PC12 cells through inhibition of RhoA/ROCK-1 signalling.. J Neurochem.

[17] Villalonga P, Guasch RM, Riento K, Ridley AJ (2004). RhoE inhibits cell cycle progression and Ras-induced transformation.. Mol Cell Biol.

[18] Poch E, Minambres R, Mocholi E, Ivorra C, Perez-Arago A (2007). RhoE interferes with Rb inactivation and regulates the proliferation and survival of the U87 human glioblastoma cell line.. Exp Cell Res.

[19] Ongusaha PP, Kim HG, Boswell SA, Ridley AJ, Der CJ (2006). RhoE is a pro-survival p53 target gene that inhibits ROCK I-mediated apoptosis in response to genotoxic stress.. Curr Biol.

[20] Liebig T, Erasmus J, Kalaji R, Davies D, Loirand G (2009). RhoE is required for keratinocyte differentiation and stratification.. Mol Biol Cell.

[21] Fortier M, Comunale F, Kucharczak J, Blangy A, Charrasse S (2008). RhoE controls myoblast alignment prior fusion through RhoA and ROCK.. Cell Death Diff.

[22] Lin L, Xu B, Rote NS (1999). Expression of endogenous retrovirus ERV-3 induces differentiation in BeWo, a choriocarcinoma model of human placental trophoblast.. Placenta.

[23] Tannetta DS, Sargent IL, Linton EA, Redman CWG (2008). Vitamins C and E inhibit apoptosis of cultured human term placenta trophoblast.. Placenta.

[24] Collett GP, Linton EA, Redman CWG, Sargent IL (2010). Downregulation of caveolin-1 enhances fusion of human BeWo choriocarcinoma cells.. PLoS ONE.

[25] Rashid-Doubell F, Tannetta D, Redman CWG, Sargent IL, Boyd CAR (2007). Caveolin-1 and lipid rafts in confluent BeWo trophoblasts: evidence for Rock-1 association with caveolin-1.. Placenta.

[26] Robinson JM, Ackerman IV WE, Behrendt NJ, Vandre DD (2009). While dysferlin and myoferlin are coexpressed in the human placenta, only dysferlin expression is responsive to trophoblast fusion in model systems.. Biol Reprod.

[27] Sands WA, Palmer TM (2008). Regulating gene transcription in response to cyclic AMP elevation.. Cell Signal.

[28] Alsat E, Wyplosz P, Malassine A, Guibourdenche J, Porquet D (1996). Hypoxia impairs cell fusion and differentiation process in human cytotrophoblast, in vitro.. J Cell Physiol.

[29] Heazell AEP, Lacey HA, Jones CJP, Huppertz B, Baker PN (2008). Effects of oxygen on cell turnover and expression of regulators of apoptosis in human placental trophoblast.. Placenta.

[30] Keryer G, Alsat E, Tasken K, Evain-Brion D (1998). Cyclic-AMP dependent protein kinases and human trophoblast cell differentiation in vitro.. J Cell Sci.

[31] Frendo JL, Olivier D, Cheynet D, Blond JL, Bouton O (2003). Direct involvement of HERV-W env glycoprotein in human trophoblast cell fusion and differentiation.. Mol Cell Biol.

[32] Chen CP, Chen LF, Yang SR, Chen CY, Ko CC (2008). Functional characterization of the human placental fusogenic membrane protein syncytin 2.. Biol Reprod.

[33] Esnault C, Priet S, Ribet D, Vernochet C, Bruls T (2008). A placenta-specific receptor for the fusogenic, endogenous retrovirus-derived, human syncytin-2.. Proc Natl Acad Sci USA.

[34] Muroi Y, Sakurai T, Hanashi A, Kubota K, Nagaoka K (2009). CD9 regulates transcription factor GCM1 and ERVWE1 expression through cAMP/protein kinase A signaling pathway.. Reproduction.

[35] Shurin GV, Tourkova IL, Shurin MR (2008). Low-dose chemotherapeutic agents regulate small Rho GTPase activity in dendritic cells.. J Immunother.

[36] Boswell SA, Ongusaha PP, Nghiem P, Lee SW (2007). The protective role of a small GTPase RhoE against UVB-induced DNA damage in keratinocytes.. J Biol Chem.

[37] Shimomura A, Ohama T, Hori M, Ozaki H (2009). 17β-estradiol induces gastrointestinal motility disorder by decreasing CPI-17 phosphorylation via changes in rho-family G-protein Rnd expression in small intestine.. J Vet Med Sci.

[38] Riento K, Totty N, Villalonga P, Garg R, Guasch R (2005). RhoE function is regulated by ROCK I-mediated phosphorylation.. EMBO J.

[39] Madigan JP, Bodemann BO, Brady DC, Dewar BJ, Keller PJ (2009). Regulation of Rnd3 localization and function by protein kinase C alpha-mediated phosphorylation.. Biochem J.

[40] Vargas A, Moreau J, Le Bellego F, Lafond J, Barbeau B (2008). Induction of trophoblast cell fusion by a tyrosine phosphatase inhibitor.. Placenta.

[41] Al-Nasiry S, Spitz B, Hanssens M, Luyten C, Pijnenborg R (2006). Differential effects of inducers of syncytialization and apoptosis on BeWo and JEG-3 choriocarcinoma cells.. Hum Reprod.

[42] Orendi K, Gauster M, Moser G, Meiri H, Huppertz B (2010). The choriocarcinoma cell line BeWo: syncytial fusion and expression of syncytium-specific proteins.. Reproduction.

[43] Schubert SW, Abendroth A, Kilian K, Vogler T, Mayr B (2008). bZIP-type transcription factors CREB and OASIS bind and stimulate the promoter of the mammalian transcription factor GCMa/GCM1 in trophoblast cells.. Nucleic Acids Res.

[44] Knerr I, Schubert SW, Wich C, Amman K, Aigner T (2005). Stimulation of GCMa and syncytin via cAMP mediated PKA signaling in human trophoblastic cells under normal and hypoxic conditions.. FEBS Lett.

[45] Chang CW, Chuang HC, Yu C, Yao TP, Chen H (2005). Stimulation of GCMa transcriptional activity by cyclic AMP/protein kinase A signaling is attributed to CBP-mediated acetylation of GCMa.. Mol Cell Biol.

[46] Chiang MH, Liang FY, Chen CP, Chang CW, Cheong ML (2009). Mechanism of hypoxia-induced GCM1 degradation: implications for the pathogenesis of preeclampsia.. J Biol Chem.

[47] Kudo Y, Boyd CAR, Sargent IL, Redman CWG (2003). Hypoxia alters expression and function of syncytin and its receptor during trophoblast cell fusion of human placental BeWo cells: implications for impaired trophoblast fusion in pre-eclampsia.. Biochim Biophys Acta.

[48] Getsios S, MacCalman CD (2003). Cadherin-11 modulates the terminal differentiation and fusion of human trophoblastic cells in vitro.. Dev Biol.

